# Ole1, fatty acid desaturase, is required for Atg9 delivery and isolation membrane expansion during autophagy in *Saccharomyces cerevisiae*

**DOI:** 10.1242/bio.022053

**Published:** 2016-11-23

**Authors:** Yuta Ogasawara, Shintaro Kira, Yukio Mukai, Takeshi Noda, Akitsugu Yamamoto

**Affiliations:** 1 Nagahama Institute of Bio-Science and Technology, 1266 Tamura, Nagahama, Shiga 526-0829, Japan; 2 Department of Anatomy and Molecular Cell Biology, Nagoya University Graduate School of Medicine, Nagoya 466-8550, Japan; 3 Graduate School of Dentistry, Osaka University, 1-8 Yamadaoka, Suita, Osaka 565-0871, Japan; 4 Graduate school of Frontier Bioscience, Osaka University, 1-8 Yamadaoka, Suita, Japan

**Keywords:** Autophagy, Unsaturated fatty acid, OLE1, Fatty acid monodesaturation

## Abstract

Macroautophagy, a major degradation pathway of cytoplasmic components, is carried out through formation of a double-membrane structure, the autophagosome. Although the involvement of specific lipid species in the formation process remains largely obscure, we recently showed that mono-unsaturated fatty acids (MUFA) generated by stearoyl-CoA desaturase 1 (SCD1) are required for autophagosome formation in mammalian cells. To obtain further insight into the role of MUFA in autophagy, in this study we analyzed the autophagic phenotypes of the yeast mutant of *OLE1*, an orthologue of SCD1. *Δole1* cells were defective in nitrogen starvation-induced autophagy, and the Cvt pathway, when oleic acid was not supplied. Defects in elongation of the isolation membrane led to a defect in autophagosome formation. In the absence of Ole1, the transmembrane protein Atg9 was not able to reach the pre-autophagosomal structure (PAS), the site of autophagosome formation. Thus, autophagosome formation requires Ole1 during the delivery of Atg9 to the PAS/autophagosome from its cellular reservoir.

## Abstract (precis)

**Summary:** Isolation membrane expansion in yeast autophagy requires mono-unsaturated fatty acids generated by Ole1, fatty acid desaturase during delivery of Atg9 to the PAS/autophagosome from its cellular reservoir.

## INTRODUCTION

Autophagy, a major degradation pathway of cytoplasmic components, affects numerous pathophysiological processes such as adaptation to starvation, prevention of neurodegeneration, and protection against bacterial invasion (Mizushima et al., 2008). Among various forms of autophagy, macroautophagy is characterized by the involvement of a double-membrane structure called the autophagosome. Autophagosomes elongate from cup-shaped membranous structures, termed isolation membrane, to enwrap targeted cytosolic components. Subsequently, they fuse with lysosomes/vacuoles, where their contents are degraded. Autophagosome formation, which involves dynamic membrane organization, has been extensively studied in recent years. The Atg proteins responsible for this process are evolutionarily conserved from yeast to human, and consequently, studies in yeast have made significant contributions to this field (Nakatogawa et al., 2009; Reggiori and Klionsky, 2013).

However, it remains unclear whether specific lipid species play a specialized role in this process. The two best-characterized lipid species involved in autophagosome formation are phosphatidylinositol 3-phosphate and phosphatidylethanolamine (Noda et al., 2002, 2010). A recent study by our group demonstrated that, in addition to these lipid species, mono-unsaturated fatty acids (MUFA) are required for autophagosome formation in mammalian cells (Ogasawara et al., 2014). Stearoyl-CoA desaturase 1 (SCD1) is the key enzyme responsible for biosynthesis of MUFA from saturated fatty acids (SFA) (Kim and Ntambi, 1999). Inactivation of SCD1 using a specific inhibitor, 28c, results in defective translocation of ULK1, a mammalian homologue of Atg1, to sites of autophagosome formation (Ogasawara et al., 2014). Additionally, Desat1, a *Drosophila* orthologue of *SCD1*, is essential for autophagy (Köhler et al., 2009). The major product of SCD1, oleic acid (OA), is incorporated into multiple types of lipids including phospholipids, triglycerides, and cholesteryl esters (Paton and Ntambi, 2009). Due to the bent structure of their alkyl chains, MUFA are proposed confer curvature into lipid bilayer (Kamal et al., 2009). In light of the bending properties of the cup-shaped isolation membrane, it is tempting to speculate that MUFA play universally specific roles in autophagy, especially autophagosome formation. Consistent with this idea, unsaturated fatty acid is enriched in the isolation membrane (Reunanen et al., 1985). The yeast *Saccharomyces cerevisiae* has one SCD1 orthologue, Ole1 (Stukey et al., 1989). In this study, we investigated whether MUFA play a role in autophagy by analyzing the yeast *Δole1* mutant and exploring its underlying mechanism.

## RESULTS AND DISCUSSION

### The fatty acid monodesaturase Ole1 is required for autophagy

**Fig. 1.**
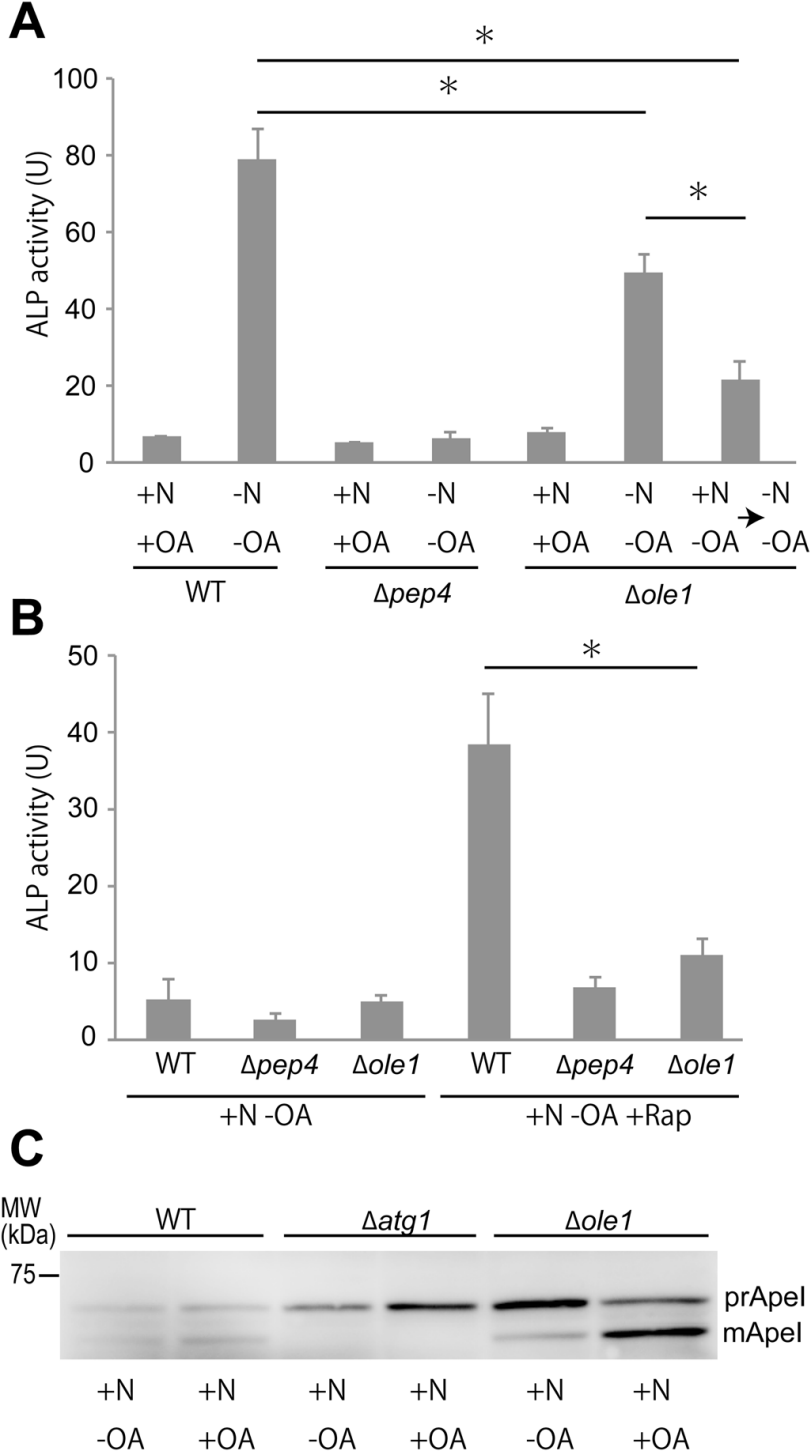
**Ole1 is required for macroautophagy and the Cvt pathway.** (A) Wild-type (SKY084), Δ*pep4* (SKY100) or Δ*ole1* (SKY84-ole1) cells were grown in YPD+OA (+N+OA), SD (-N) without OA for 3 h (-N-OA), or pre-incubation in YPD without OA for 2 h followed by SD(-N) without OA for 3 h (+N-OA→-N-OA). Cells were then subjected to ALP assay. ALP activity was defined as emission per mass of protein in the reaction (μg) and reaction time (min). Data represent means±s.e. of three independent experiments. *P<0.01 of unpaired two-tailed Student's *t*-test. (B) Wild-type (SKY084), Δ*pep4* (SKY100), or Δ*ole1*(SKY84-ole1) cells were grown in YPD+OA, incubated in YPD without OA for 2 h (+N-OA), further incubated in YPD with 500 nM rapamycin (+N-OA+Rap), and then subjected to ALP assay. Data represent means±s.e. of three independent experiments. *P<0.01 of unpaired two-tailed Student's *t*-test (C) Wild-type (BY4741), Δ*atg1* (EUROSCARF), or Δ*ole1* (BY4741-ole1) cells were grown in YPD+OA (+N+OA), or in YPD-OA for 5 h (+N-OA). Cell lysates were processed for immunoblot analysis to detect precursor Ape1 (prApe1) and mature Ape1 (mApe1).

To determine whether fatty acid desaturation plays a role in yeast autophagy, we analyzed the deletion mutant of *OLE1*, which encodes the Δ9 fatty acid desaturase in yeast (Stukey et al., 1989). To this end, we first measured autophagic activity using the alkaline phosphate (ALP) assay (Noda and Klionsky, 2008). Activity of artificially engineered ALP (alkaline phosphatase, Pho8Δ60), reflecting autophagic activity, increased under nitrogen starvation in wild-type cells, but not in *Δpep4* cells lacking proteinase A, which is essential for autophagic degradation (Fig. 1A). Growth of *Δole1* cells requires supplementation with 1 mM oleic acid (hereafter OA) in the medium (Fig. S1A,B) (Stukey et al., 1989). When *Δole1* cells were transferred from rich medium containing OA directly into nitrogen starvation medium without OA, they exhibited a partial defect in autophagy (Fig. 1A). However, when they were pre-incubated in rich medium without OA for 2 h, thus titrating out the intracellular OA pool (Stukey et al., 1989), ALP activity was significantly decreased (Fig. 1A). Thus, Ole1 is required for starvation-induced macroautophagy. Starvation-induced macroautophagy is caused by inactivation of TORC1; accordingly, treatment with rapamycin, a TORC1-specific inhibitor, also induces autophagy (Noda and Ohsumi, 1998). This induction was defective in *Δole1* cells lacking OA (Fig. 1B), indicating that the defect in *Δole1* is downstream of TORC1.

Next, we monitored the cytoplasm-to-vacuole targeting (Cvt) pathway, a constitutive selective autophagy that shares many components involved in macroautophagy (Scott et al., 1996). The mature form of ApeI, the cargo of the Cvt pathway, was not observed in *Δatg1* cells, indicating that the Cvt pathway is defective in this mutant (Fig. 1C) (Scott et al., 1996). In *Δole1* cells, mature ApeI was present in the OA-supplemented condition; however, when the cells lacked OA, the pro-form of Ape1 accumulated, indicating that Ole1 is also required for the Cvt pathway (Fig. 1C). Therefore, Ole1 deficiency interferes with a step of autophagy that is shared by starvation-induced macroautophagy and the Cvt pathway, rather than in a signaling process involved in the induction of autophagy.

### The *Δole1* mutant is defective in membrane elongation during autophagosome formation

**Fig. 2.**
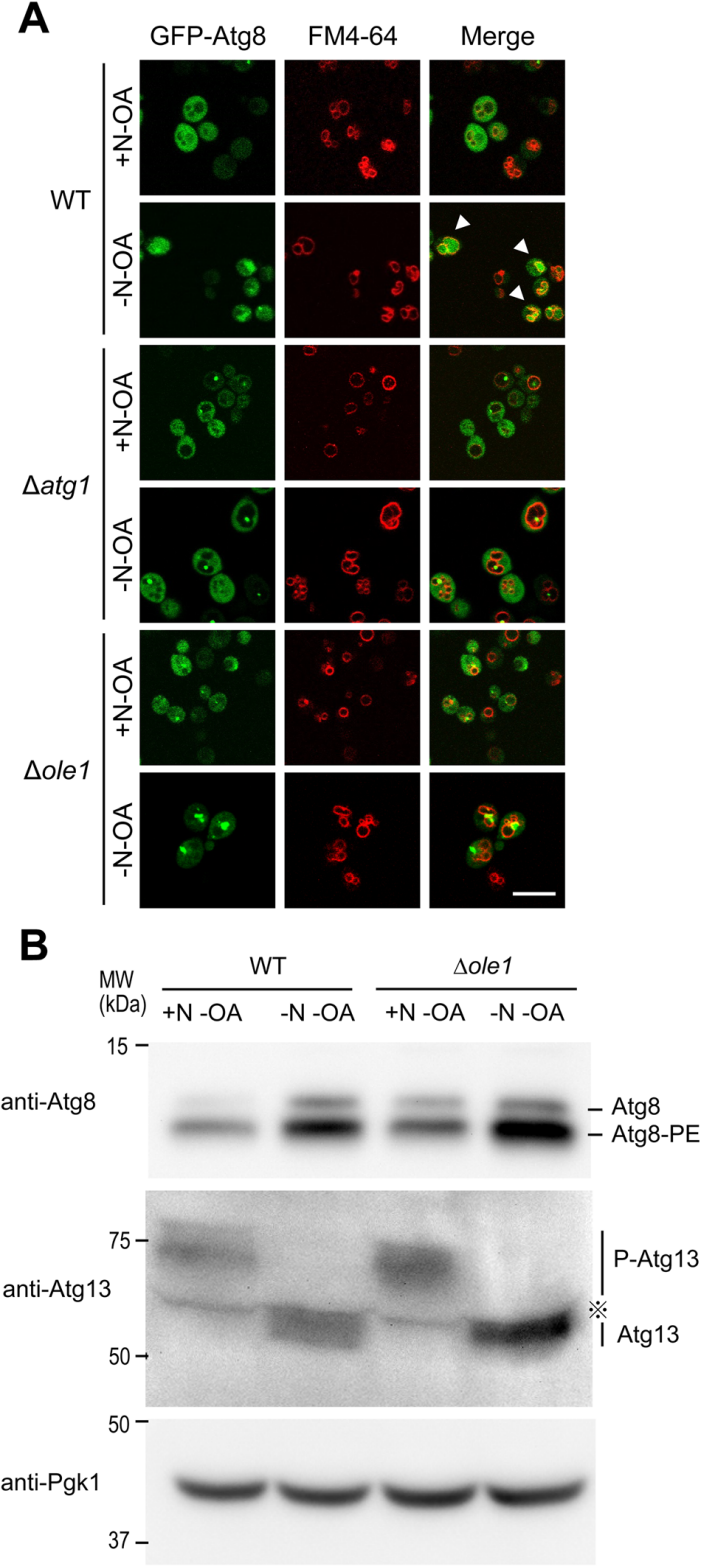
**GFP-Atg8 translocation into the vacuole via autophagy was suppressed in the *Δole1* mutant.** (A) Wild-type (BY4741), Δ*atg1* (EUROSCARF), or Δ*ole1* cells (BY4741-ole1) harboring pRS316/GFP-Atg8 were grown in SC-Ura+OA, shifted to SC-URA-OA for 2 h (+N-OA), and incubated further in SD-N without OA for 3 h (-N-OA). Cells were observed under a fluorescence microscope. Scale bars: 10 μm. (B) Wild-type (BY4741) or Δ*ole1* cells (BY4741-*ole*1) were grown in YPD+OA, shifted to YPD without OA (+N-OA) for 2 h, and further incubated in SD-N medium for 1 h (-N-OA). Cell lysates were processed for immunoblot analysis to detect Atg13, Atg8, and Pgk1 (used as an endogenous control).

To determine which step in autophagy was affected, we examined the behavior of the Atg8 protein, which is essential for autophagosome formation. Atg8 proteins are trapped inside autophagosomes during autophagosome formation, and can therefore be used to track this process (Kirisako et al., 1999). In wild-type cells, fluorescent signals derived from GFP-Atg8 were observed in the vacuolar lumen under starvation conditions, reflecting the normal progression of autophagy (Fig. 2A) (Kirisako et al., 1999). If the fusion step between autophagosome and vacuole is defective, fluorescent puncta should accumulate in the cytoplasm (Ishihara et al., 2001), whereas if the autophagic body degradation is defective, a punctate signal should appear inside the vacuole (Kirisako et al., 1999). However, *Δole1* mutants exhibited significantly reduced vacuolar luminal signals, similar to those of *Δatg1*, which are defective in autophagosome formation (Fig. 2A). This could also happen if conjugation of Atg8 with phosphatidylethanolamine (PE) is defective (Ichimura et al., 2000; Kirisako et al., 2000); however, Atg8-PE formation occurred normally in *Δole1* in the absence of OA (Fig. 2B). These data suggest that autophagosome formation is defective in the *Δole1* mutant.

**Fig. 3.**
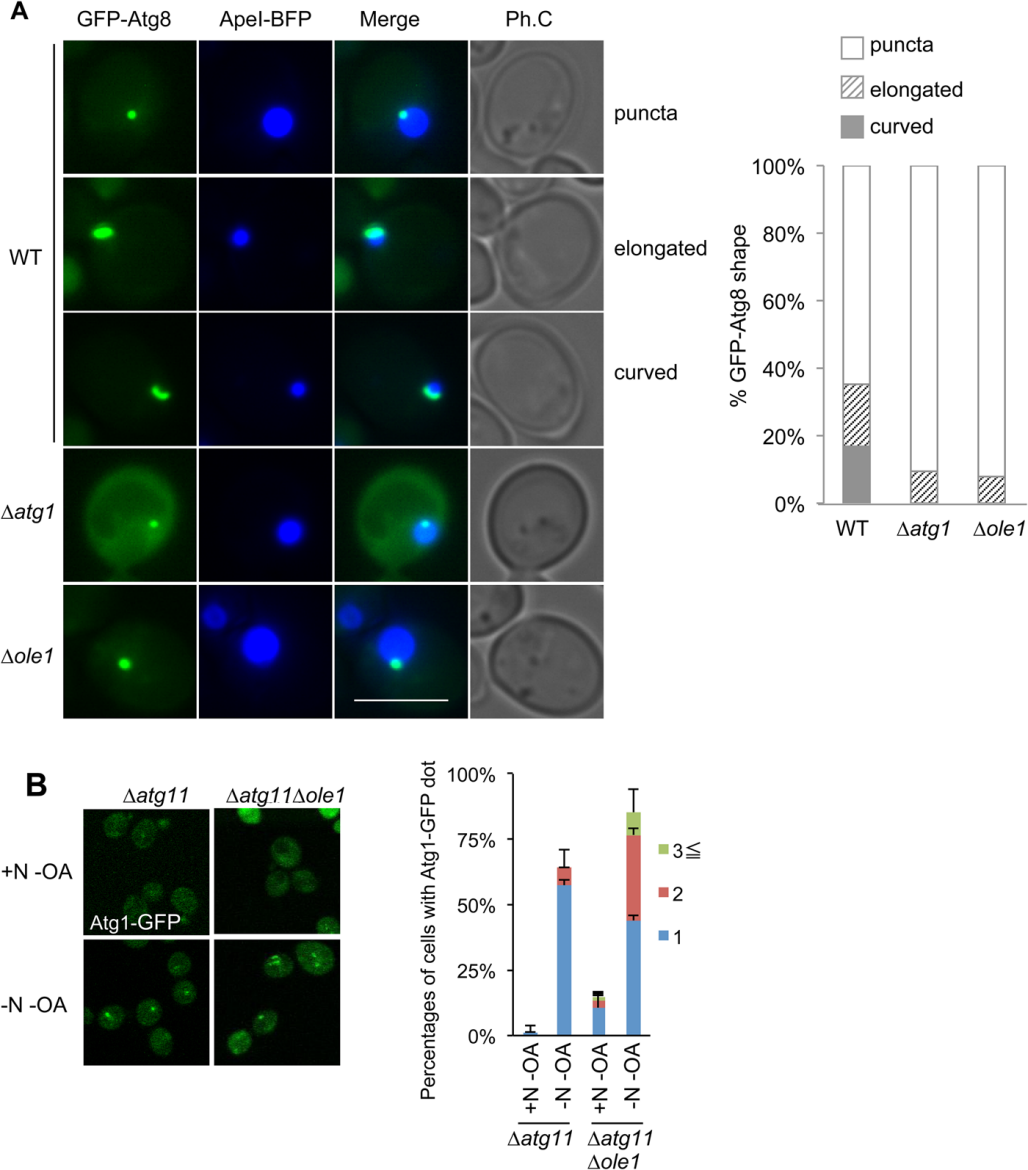
**Isolation membrane formation is defected in *ole1* mutants.** (A) Wild-type (SKY462-A), Δ*atg1* (SKY466-A), or Δ*ole1* (SKY462-A Δ*ole1*::*hphNT1*) cells harboring pSK399 (pRS426/pCup1-ApeI-EBFP) were grown in SCD+OA containing 250 µM CuS0_4_ for 8 h, transferred to SCD medium without OA with 0.4 μg/ml rapamycin, and incubated for 6 h. Cells were then observed by microscopy. Scale bars: 5 μm. Quantification of elongated, curved, or punctate structures containing of GFP-Atg8 is shown in graphs. Approximately 50 cells were counted for each strain. (B) Δ*atg11* (SKY157) or Δ*atg11* Δ*ole1* (SKY157-*ole1*) cells expressing GFP-tagged Atg1 were grown in YPD+OA, shifted to YPD without OA for 2 h (+N-OA), and further incubated in SD-N without OA for 3 h (-N-OA). Cells were observed under a fluorescence microscope. Scale bars: 10 μm. Percentages of cells with one, two, or three or more Atg1-GFP dot(s) per cell are shown in the graph. Data represent means±s.e. of three independent experiments, in each of which more than 50 cells were counted.

Therefore, we next investigated whether the autophagosome formation process is indeed perturbed in the *Δole1* mutant using a system that enables visualization of the membrane elongation process (Suzuki et al., 2013). When ApeI is overproduced, it forms giant aggregates in the cytoplasm that cannot be entirely surrounded by autophagosome membrane; consequently, the enwrapping process remains incomplete. In wild-type cells, in addition to a punctate PAS pattern, we frequently observed a curved pattern of GFP-Atg8 in association with a giant ApeI-EBFP complex (Fig. 3A, ‘curved’), as well as an elongated but not curved pattern (Fig. 3A, ‘elongated’). In *Δatg1* cells, which are defective in elongation, only a few cells had this elongated pattern, and no cells with the curved pattern were observed (Fig. 3A) (Suzuki et al., 2013). Likewise, *Δole1* cells rarely exhibited the elongated pattern, and never had the curved pattern (Fig. 3A). These data indicate that elongation of membrane during autophagosome formation is defective in the *Δole1* mutant.

### Atg9 traffic to the PAS is defective in the *Δole1* mutant

To explore the molecular mechanism underlying this defect, we asked whether Atg1 protein kinase is recruited to the PAS, where autophagosome formation occurs (Suzuki et al., 2001). To strictly investigate starvation-dependent PAS recruitment, we knocked out the *ATG11* gene to exclude starvation-independent PAS recruitment (Kawamata et al., 2008). In wild-type (but *Δatg11*) cells, GFP-Atg1 formed puncta only under starvation, representing PAS formation (Fig. 3B) (Kawamata et al., 2008). In *Δole1* cells, formation of GFP-Atg1 puncta was also observed, although more puncta were present (Fig. 3B). Atg1 constitutes the scaffold/initiation complex of the PAS along with At13, Atg17, Atg29 and Atg31, and these puncta are likely to represent this complex (Yamamoto et al., 2016). Formation of this complex is regulated by dephosphorylation of Atg13, and this process occurred normally in the *Δole1* mutant, just as in wild-type cells (Fig. 2B) (Yamamoto et al., 2016).

**Fig. 4.**
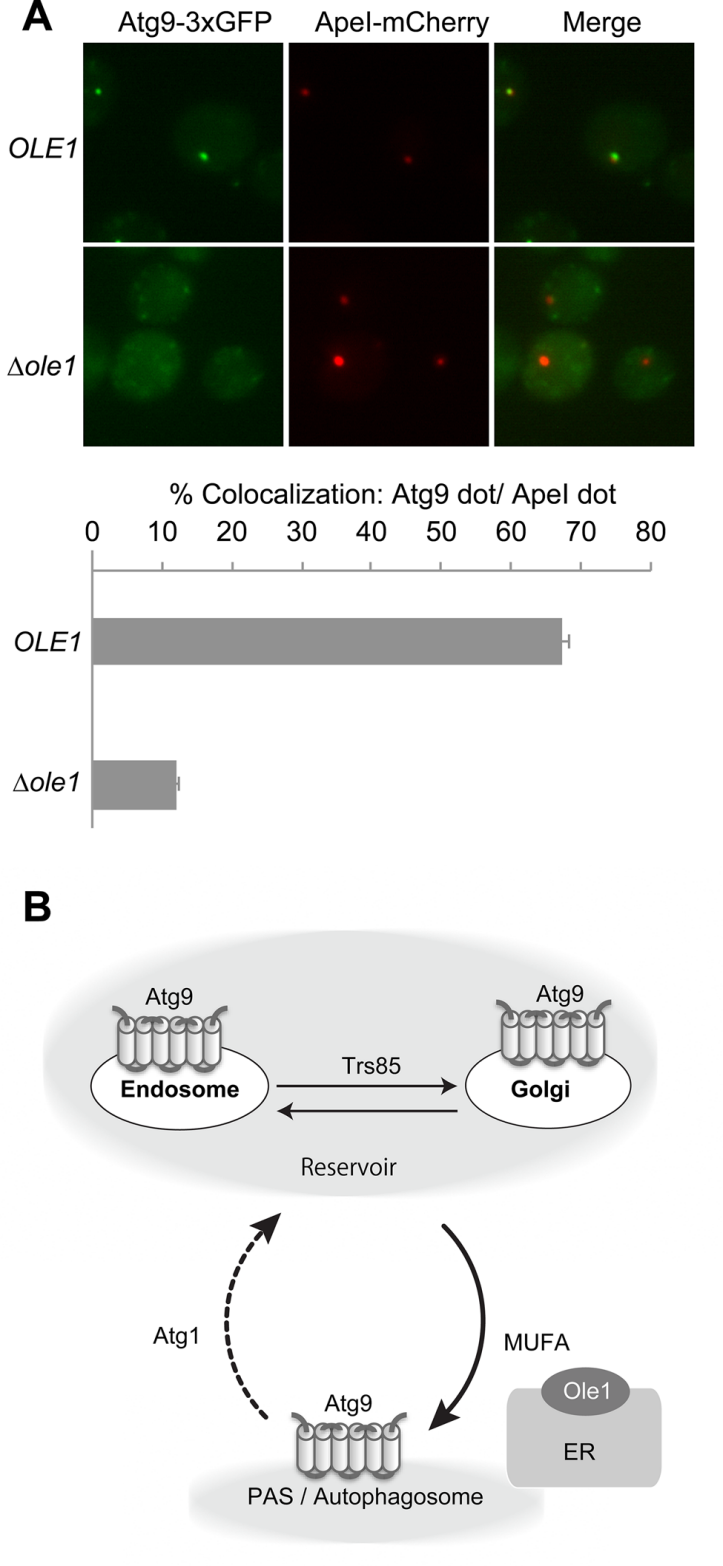
**Ole1 is required for Atg9 trafficking toward PAS.** (A) KNY076 (*atg9::ATG9-3xGFP:HIS3*, Δ*atg1::kanMX6*, *Trs85-IAA17-3HA::hphNT1*, *OsTIR1-9xmyc:URA3*), and SKY569 (KNY076, Δ*ole1::natNT2*) cells harboring pRS314/ApeI-mCherry were grown overnight in SCD+OA and 500 µM NAA, and then passaged in the same medium and cultured for an additional 8 h. Cells were washed three times with SCD containing 500 µM NAA without OA, resuspended, and cultured for 2 h. Cells were washed twice with SCD media, resuspended in SCD without OA containing 200 ng/ml rapamycin, and cultured for 2 h. The cells were then subjected to microscopy. Cells with ApeI-mCherry dot colocalized with Atg9-3xGFP dot were counted; percentages are shown. A total of 100–150 cells were counted for the calculation. Data represent means and standard deviation of three independent experiments. (B) Model depicting the conclusions of this study. Atg9 cycles between the PAS/autophagosome and reservoir (endosome and Golgi). In the absence of MUFA in the *Δole1* mutant, Atg9 cannot reach the PAS. Thus, MUFA is required for this traffic.

Finally we examined the behavior of Atg9, a transmembrane protein essential for autophagosome formation (Noda et al., 2000). Atg9 shuttles between two populations, the PAS/autophagosome and reservoir fractions; in *atg1* mutants, Atg9 cannot be retrieved from the PAS and consequently accumulated there (Reggiori et al., 2004). We proposed that the Atg9 reservoir consists of the vesicles trafficking between the Golgi and endosome (Shirahama-Noda et al., 2013). In the mutant of *trs85*, a specific subunit of transport protein particle (TRAPP III), Atg9 is not delivered to the PAS from the reservoir especially under nutrient-rich conditions (Kakuta et al., 2012; Shirahama-Noda et al., 2013). To examine the effect of OA deficiency on Atg9 traffic, we needed to segregate these anterograde and retrograde pathways; to this end, we applied the conditional degron system (AID) to control the expression of Trs85, which worked well in previous studies (Nishimura et al., 2009; Shirahama-Noda et al., 2013). Addition of 1-Naphthaleneacetic acid (NAA) results in rapid degradation of the endogenous Trs85 protein tagged with IAA by the ubiquitin-proteasome system (Nishimura et al., 2009; Shirahama-Noda et al., 2013). Under nutrient-rich conditions in the presence of OA and NAA, Trs85 expression is suppressed due to degradation, so that Atg9 cannot reach PAS and does not accumulate there, even in the *Δatg1* mutant (Shirahama-Noda et al., 2013). After 2 h of pre-incubation with NAA but without OA under nutrient-rich conditions, to titrate out the intracellular OA pool, the cells were treated with rapamycin without OA and without NAA to recover expression of Trs85 (Shirahama-Noda et al., 2013). In *OLE1* wild-type cells, Atg9-3xGFP efficiently accumulated at PAS (Fig. 4A), whereas in *Δole1* cells Atg9-3xGFP could not reach the PAS (Fig. 4A). Atg9 plays an important role in elongation of the isolation membrane (Suzuki et al., 2013). Thus, the defect in membrane elongation in *Δole1* cells could be attributed to the defect in delivery of Atg9 to the PAS. The elevated number of Atg1 puncta in *Δole1* cells (Fig. 3B) might be attributed to the lower supply of Atg9 to the PAS and resultant hyper-formation of the scaffold complex; a similar increment is also observed in *Δatg9* cells (Suzuki et al., 2007).

When saturated fatty acids accumulate in yeast, the late secretory pathway from the trans-Golgi network is defective, and ER morphology is altered (Payet et al., 2013; Pineau and Ferreira, 2010). However, traffic of some cargo proteins, such as plasma membrane ATPase Pma1 and invertase, are not affected under these conditions (Pineau and Ferreira, 2010). We examined the effect on traffic of a typical marker protein, carboxypeptidase Y, which travels through ER and Golgi to the vacuole (Robinson et al., 1988). Even under starvation conditions, when expression of this protein was up-regulated, its pro-form did not accumulate in *Δole1* cells, suggesting that membrane traffic is not universally affected (Fig. S2). Further studies will be required to completely elucidate the process involved in Atg9 traffic. In particular, the step from the reservoir to PAS is incompletely understood. Recently, the ER was proposed to be closely linked to the autophagosome formation not only in mammalian cells, but also in yeast (Graef et al., 2013; Hayashi-Nishino et al., 2009). During autophagosome formation, Atg9 resides at the edge of the isolation membrane, in close proximity to the ER exit site (ERES) (Suzuki et al*.*, 2013). Ole1 resides in the ER, and its localization is not altered by nitrogen starvation conditions (Fig. S3) (Tatzer et al., 2002). One possible model is that Atg9 is delivered from the reservoir (Golgi/endosome) to the ER, and then to the isolation membrane; if so, MUFA might contribute to one or more steps during this process (Fig. 4B). In quantitative terms, it is possible that the amount of membrane derived from Atg9-containing vesicles in the reservoir is too small to supply the whole autophagosome membrane (Yamamoto et al., 2012); however, it remains possible that MUFA is supplied to the autophagosome membrane along with Atg9 traffic. Further dissection of these processes should elucidate the contribution of Ole1 to autophagy.

## MATERIALS AND METHODS

### Yeast culture

Yeast cells were grown in YPD medium (1% yeast extract, 2% peptone, 2% glucose), SD-Ura (0.67% yeast nitrogen base and 2% glucose supplemented with amino acids without uracil), or SCD (0.67% yeast nitrogen base, 2% glucose, 0.5% casamino acids) at 30°C. Oleic acid (Nacalai Tesque, Kyoto, Japan, 25630-51) at a final concentration of 1 mM and 1% (v/v) Triton X-100 was added to support growth of the *Δole1* mutant (Stukey et al., 1989). To induce autophagy, cells were incubated in SD-N medium (0.17% yeast nitrogen base without ammonium sulfate and amino acids, and 2% glucose) or YPD medium containing 200 ng/ml rapamycin (LKT Biolaboratories, MA, USA, R0161) with or without OA. 1-Naphthaleneacetic acid (NAA) (Sigma-Aldrich, N0640) at 500 µM was added to induce degradation of Trs85 (Shirahama-Noda et al., 2013).

### Yeast strains and plasmid construction

The yeast strains used in this study are listed in Table S1. All deletion strains and genomically tagged strains were constructed by standard gene targeting methods (Janke et al., 2004; Longtine et al., 1998). pSK399, which harbors pCup1-ApeI-EBFP in pRS426, was generated as follows. The promoter region of *CUP1-1* was PCR-amplified from plasmid pYM-N2 (Janke et al., 2004), digested with *Sac*I and *Not*I, and cloned into pRS426 (pSK397). The ApeI-mCherry fragment was amplified from mCherry-tagged yeast ApeI in genomic DNA, digested with *Not*I and *Cla*I, and cloned into pSK397 (pSK398). The inverse PCR product of pSK398 lacking the mCherry region, and the EBFP ORF DNA fragment amplified from pYM35 (Janke et al., 2004), were co-transformed into BY4741 (Brachmann et al., 1998) for GAP-repair cloning to generate pSK399 (Kitazono, 2011).

### ALP assay

Autophagic activity was measured by ALP assay as reported (Noda and Klionsky, 2008).

### Immunoblotting

Immunoblotting was performed as reported (Kira et al., 2014; Nakatogawa et al., 2007) using rabbit anti-Ape1, anti-Atg13, anti-Atg8, anti-CPY (a gift from Dr Y. Ohsumi, Tokyo Institute of Technology, Japan), and anti-Pgk1 (Life Technologies, 459250) as primary antibodies.

### Microscopy

Cells expressing GFP-Atg8 or GFP-Atg1 were observed under a FLUOVIEW FV1000 and edited using the Olympus FV10-ASW software (Olympus, Tokyo, Japan). For visualization of IM and Atg9-3xGFP, yeast cells were observed on a Leica AF6500 fluorescence imaging system (Leica Microsystems) mounted on a DMI6000 B microscope (HCX PL APO 100/NA=1.40-0.70, oil-immersion objective lens, xenon lamp; Leica Microsystems) under the control of the LAS-AF software (Leica Microsystems).
